# Wavefront Restoration Technology of Dynamic Non-Uniform Intensity Distribution Based on Extreme Learning Machine

**DOI:** 10.3390/s21113877

**Published:** 2021-06-04

**Authors:** Haiqi Lin, Xing He, Shuai Wang, Ping Yang

**Affiliations:** 1 Key Laboratory on Adaptive Optics, Chinese Academy of Sciences, Chengdu 610209, China; hexingjiayou@126.com (X.H.); wangshuai@ioe.ac.cn (S.W.); pingyang2516@163.com (P.Y.); 2 Institute of Optics and Electronics, Chinese Academy of Sciences, Chengdu 610209, China

**Keywords:** non-uniform intensity, the wavefront sensor, wavefront restoration, the extreme learning machine method, modal algorithm

## Abstract

Non-uniform intensity distribution of laser near-field beam results in the irregular shape of the spot in the wavefront sensor. The intensity of some sub-aperture spots may be too weak to be detected, and the accuracy of wavefront restoration is seriously affected. Therefore, an extreme learning machine method is proposed to realize high precision wavefront restoration under dynamic non-uniform intensity distribution. The simulation results show that this method has better accuracy of wavefront restoration than the classical modal algorithm under dynamic non-uniform intensity distribution. The root mean square error of the residual wavefront for the proposed method is only 2.9% of the initial value.

## 1. Introduction

**Figure 1:**
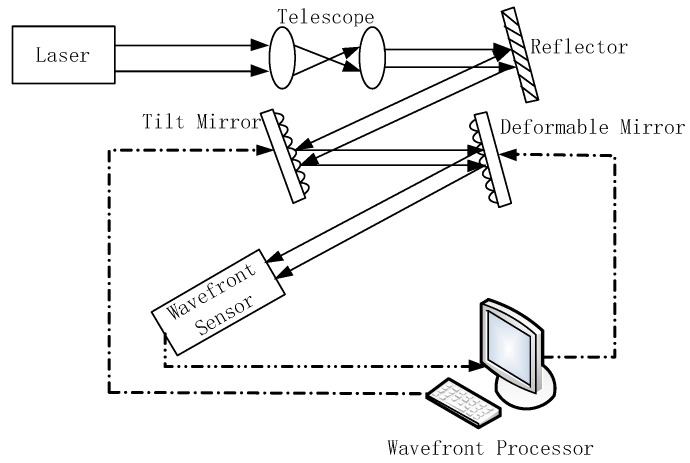
Schematic diagram of adaptive optics.

At present, many laser applications require lasers to not only produce high-power output beam, but also maintain high beam quality [1,2,3,4]. Unfortunately, as the laser power continues to increase, the thermal effect of the laser not only limits the improvement of the beam quality severely, but also makes the beam diameter and beam divergence change greatly [5,6]. How to obtain high average power and high beam quality at the same time is a basic physical problem in laser research [7,8,9]. Adaptive optics (AO) is a technology that can correct the wavefront distortion of the laser beam in real-time, as shown in Figure 1. It can compensate the dynamic laser beam aberration in real-time, and has been applied in many laser systems with remarkable results [10,11,12].

**Figure 2:**
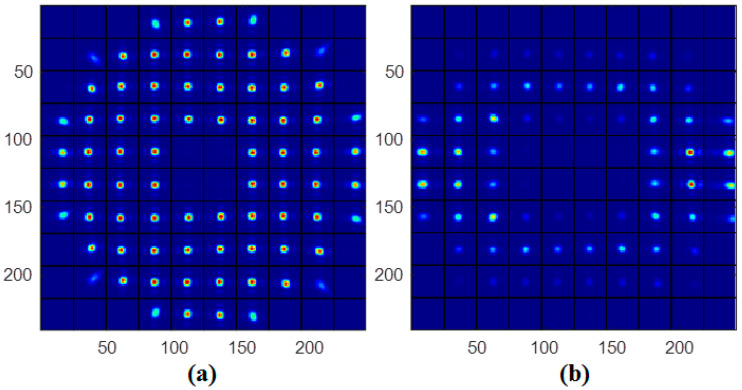
(**a**) The standard sub-aperture image of WFS. (**b**) The sub-aperture image of WFS with non-uniform near-field and irregular spots.

To correct the laser beam aberration, a wavefront sensor (WFS) is needed to detect the accurate wavefront aberration in real-time. However, with the continuous improvement of laser power and energy density, the transmission medium, and emitted light are seriously affected by the thermal effect [13,14]. It makes the intensity distribution of the laser present a dynamic non-uniform distribution. Due to the non-uniform intensity distribution of the laser beam, some sub-aperture light spots of WFS have an irregular shape or, weak intensity, or being too weak to be detected by WFS, as seen in Figure 2. The result is that WFS will lose part of the phase information, making it difficult for WFS to detect the accurate beam aberration information. This, in turn, makes it difficult for the AO system to improve the beam quality of the laser. Therefore, accurate wavefront restoration from the phase information measured by WFS under the condition of the non-uniform intensity distribution will greatly improve the beam quality for the AO system.

Recently, machine learning has been utilized to implement an image-based wavefront estimation. Xu applied the method of an extreme learning machine to accomplish the wavefront reconstruction of WFS with insufficient sub-aperture [15]. Barwick proposed the method of neural network post-processing to improve the wavefront reconstruction accuracy [16]. Guo et al., applied the artificial neural networks to estimate the Zernike coefficients and compared the wavefront reconstruction results with traditional approaches [17]. Hu et al., employed a conventional neural network (CNN) model to achieve high-order wavefront aberration detection [18]. Li et al., applied artificial neural networks to improve the robustness of SHWS in extreme situations [19]. Although the mentioned methods provide some new approaches for WFS to predict accurate Zernike coefficients, the influence of the dynamic non-uniform intensity distribution on the WFS detection accuracy, however, has not been considered and studied. Due to the non-uniform intensity distribution of the laser beam, it is difficult for WFS to detect accurate centroid displacements.

This paper mainly focuses on the advantages of machine learning methods on wavefront restoration under the dynamic non-uniform intensity distribution. When the intensity of the laser presents dynamic non-uniform distribution, some sub-aperture light spots of WFS may have an irregular shape or weak intensity. The linear regression relationship between Zernike mode coefficients of the wavefront and the local slopes measured by WFS is no longer well satisfied. In this situation, it is difficult for the classical modal algorithm based on Zernike polynomials to restore accurate Zernike mode coefficients. This paper proposes the wavefront restoration technology based on the extreme learning machine (ELM) method to construct this complicated corresponding relationship under the dynamic non-uniform intensity distribution. Compared with other gradient descent-based learning methods (such as the backpropagation algorithm), the ELM method only needs the generalized inverse matrix to obtain network weights in this case. What is more, it boasts faster training speed and better generalization performance [20,21,22]. This article will verify the accuracy of the proposed method through numerical simulations. The ELM model can restore Zernike mode coefficients more accurately than the classical modal algorithm under the non-uniform intensity distribution.

This paper is organized as follows: The ELM method’s concepts and modeling for wavefront restoration are introduced in Section 2. Section 3 shows the numerical simulation comparison results of the wavefront restoration between the proposed ELM model and the classical modal algorithm. Finally, Section 4 provides the conclusion.

## 2. Principle

**Figure 3:**
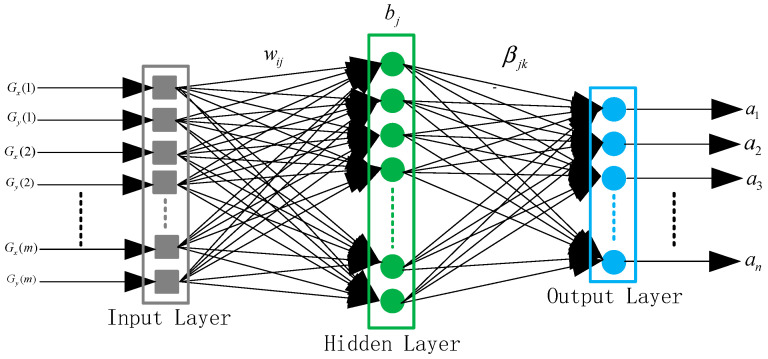
The network structure of the ELM model.

This paper will construct the ELM model to solve the problem of the linear regression relationship between Zernike mode coefficients of the wavefront and the local slopes measured by WFS under the non-uniform intensity distribution. The network structure of the ELM model is shown in Figure 3. The ELM model transmits the slope information *G* = {*G_x_*(1), *G_y_*(1), …, *G_x_*(*m*), *G_y_*(*m*)} as the input signal (input layer) to the output layer forward through the hidden layer. The output layer of the ELM model represents Zernike mode coefficients *a* = {*a*_1_, *a*_2_, …, *a_n_*} of the incident wavefront with the non-uniform intensity distribution. The slope information *G* of the incident wavefront is measured by WFS under the non-uniform intensity distribution.

According to Figure 3, the variable *w_ij_* is defined as the connection weights between the *i*-th neuron of the input layer and the *j*-th neuron of the hidden layer. *b_j_* is defined as the threshold of the *j*-th neuron of the hidden layer, and *β_jk_* is defined as the connection weights between the *j*-th neuron of the hidden layer and the *k*-th neuron of the output layer. The neural network structure of the ELM model is mathematically modeled as [23]

(1) $$a_{k}=\sum_{j=1}^{N}\beta_{jk}g(w_{ij}G_{i}+b_{j})\begin{array}{cc} & i=1,2,\ldots 2m, \end{array}\begin{array}{cc} & k=1,2,\ldots n \end{array}$$

where *m*, *N*, and *n*, respectively, represent the number of the nodes in the input layer, the number of neurons in the hidden layer, and the number of the nodes in the output layer. *g*(*x*) represents the activation function of the hidden layer neuron.

The ELM model training firstly generates random data *w_ij_* and *b_j_*. The hidden layer output matrix *H* is as follows,

(2) $$H={\left[\begin{array}{cccc} g(w_{11}G_{1}+b_{1}) & g(w_{12}G_{1}+b_{2}) & \cdots & g(w_{1N}G_{1}+b_{N})\\ g(w_{21}G_{2}+b_{1}) & g(w_{22}G_{2}+b_{2}) & \cdots & g(w_{2N}G_{2}+b_{N})\\ \vdots & \vdots & \cdots & \vdots \\ g(w_{2m1}G_{2m}+b_{1}) & g(w_{2m2}G_{2m}+b_{2}) & \cdots & g(w_{2mN}G_{2m}+b_{N}) \end{array}\right]}_{2m\times N}$$

Next, according to Equation (1), the output of the ELM model can be calculated as the least square solution of the equation *Hβ* = *a*, where *β* is the weight matrix between the hidden layer and the output layer, and *a* is the output matrix. Therefore, the weight matrix *β* can be obtained by the following equation,

(3) $$\beta =H^{†}a$$

where *H*^†^ = (*H*^T^*H*)^−1^*H*^T^ is the generalized inverse matrix of the hidden layer’s output matrix *H*.

The wavefront restoration algorithm based on the ELM model can be divided into two parts: The ELM model training and the ELM model testing. In the model training phase, the parameters of the ELM model can be obtained according to Equations (2) and (3). In order to verify the accuracy of the trained ELM model, the slope information of the test wavefront with dynamic non-uniform intensity distribution is imported into the trained ELM model. The output of the trained ELM model is Zernike mode coefficients of the test wavefront. The test wavefront with the dynamic non-uniform intensity distribution can be described as,

(4) $$\begin{array}{l} U(x,y)=Ae^{i\phi (x,y)}\\ \phi (x,y)=\sum_{i=1}^{n}a_{i}Z_{i}(x,y) \end{array}$$

where *A* is the amplitude (*A*^2^ is light intensity), *Z_i_* is the *i*-th Zernike polynomial, and *a_i_* is the corresponding Zernike mode coefficient.

## 3. Numerical Simulations

### 3.1. Dataset

A series of simulations are carried out to validate the wavefront restoration ability of the ELM model under the dynamic non-uniform intensity distribution. The key parameters of the simulations are shown in Table 1. The set of the incident wavefront with the dynamic non-uniform intensity distribution can be generated by the linear relationship of the first 15 orders of Zernike polynomials. Its slope information, which is used as the input of the ELM model training, can be measured by WFS. And the overall tilt term *a*_1_ and the piston term *a*_2_ has been removed from the slope information in the process of data measurement and acquisition. Zernike mode coefficients as the output of the ELM model training are generated by the Kolmogorov atmosphere turbulence model [24].

In order to enrich the datasets, 10,000 groups of wavefronts with the dynamic non-uniform intensity distribution have been randomly generated by employing 100 kinds of light intensity distributions measured experimentally according to the first 15 orders of Zernike polynomials and Equation (4). The order of the dataset is randomly shuffled, and 9000 groups are selected as the training set and the remaining 1000 groups as the test set.

### 3.2. Model Training and Optimization

The predictive accuracy of the ELM model is evaluated by mean square error (MSE) of Zernike mode coefficients, which is defined as follows,

(5) $$\mathrm{MSE}=\frac{\sum_{i=3}^{15}{\left(y_{i}-a_{i}\right)}^{2}}{13}$$

where *y_i_* and *a_i_* are, respectively, the target value and predicted value of the *i*-th Zernike mode coefficient (excluding the tilt term *a*_1_ and the piston term *a*_2_). The smaller MSE is, the higher the predictive accuracy of the ELM model will be obtained. When the number of output and input is given, the different activation functions in the hidden layer will affect the prediction accuracy and the number of neurons in the hidden layer. The number of hidden neurons in the ELM model is the predetermined parameter that affects the prediction accuracy. Therefore, we have analyzed the influence of different activation functions in the hidden layer on the wavefront restoration prediction accuracy for the ELM model. Table 2 shows the influence of several activation functions on the predictive accuracy of the ELM model.

According to Table 2, the proposed ELM model with the Softplus activation function can achieve the smallest MSE under the same test set.

**Figure 4:**
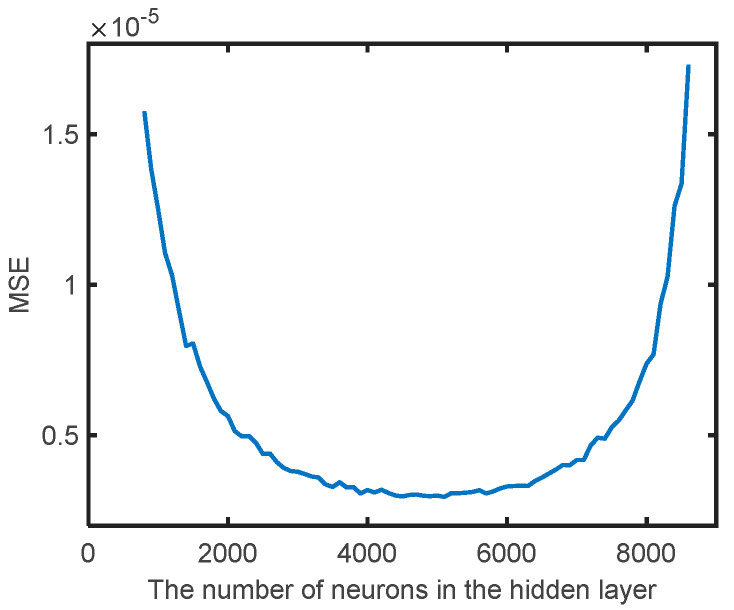
The predictive accuracy of the ELM model under different numbers of neurons in the hidden layer.

In order to determine the number of neurons in the hidden layer, the mean MSE value of 1000 test samples under a different number of neurons is shown in Figure 4. With the increasing number of neurons in the hidden layer, the predictive accuracy of the ELM model increases first and then decreases. The highest predictive accuracy of the ELM model can be obtained when the number of neurons in the hidden layer is 5100.

In conclusion, the final optimization results of the trained ELM model are as follows: The number of neurons in the hidden layer is 5100, and the activation function is selected as the Softplus activation function.

### 3.3. Prediction Results

**Figure 5:**
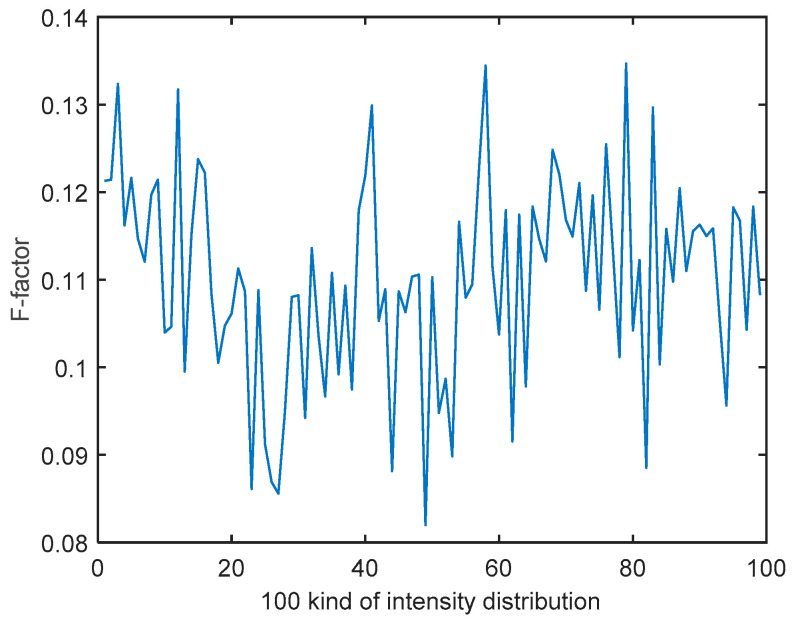
F-factor of 100 kinds of intensity distribution.

The performance of the ELM model is evaluated by a series of simulations in this section. We randomly select a set of light intensity distributions from 100 kinds of dynamic non-uniform intensity distributions, and use Equation (4) to generate a random wavefront. In this paper, F-factor is used to express the statistical characteristics of 100 kinds of intensity distribution, which is defined as follows:

(6) $$F=\frac{I_{mean}}{I_{max}}$$

where *I_mean_* represents the mean value of intensity distribution, and *I_max_* represents the maximum value of intensity distribution. F-factor of 100 kinds of intensity distribution is shown in Figure 5. There is little relevant literature or theory for reference about what intensity distribution variation so that WFS does not work properly. According to the statistical characteristics of intensity distribution, it is difficult to detect accurate wavefront aberrations for WFS in this paper when F-factor is less than 0.1347.

**Figure 6:**
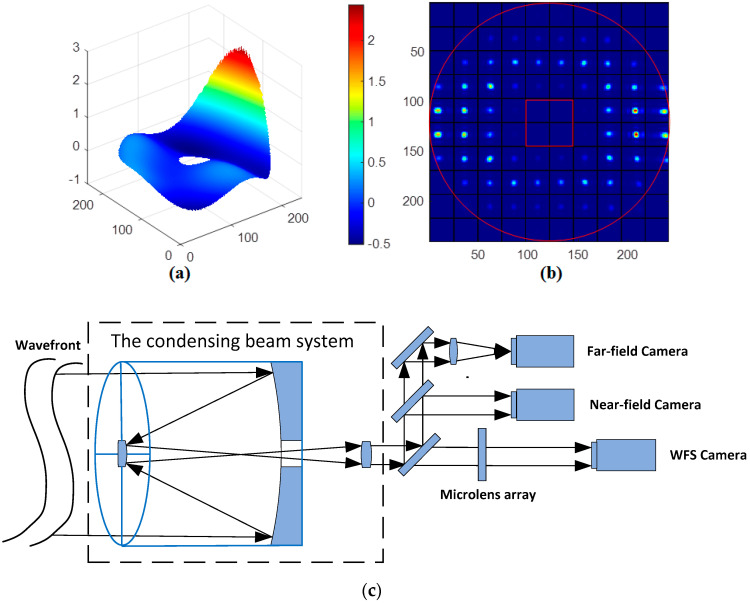
(**a**) Random wavefront phase distribution, (**b**) the sub-aperture light spot image of WFS, (**c**) WFS with the Cassegrain structure.

**Figure 7:**
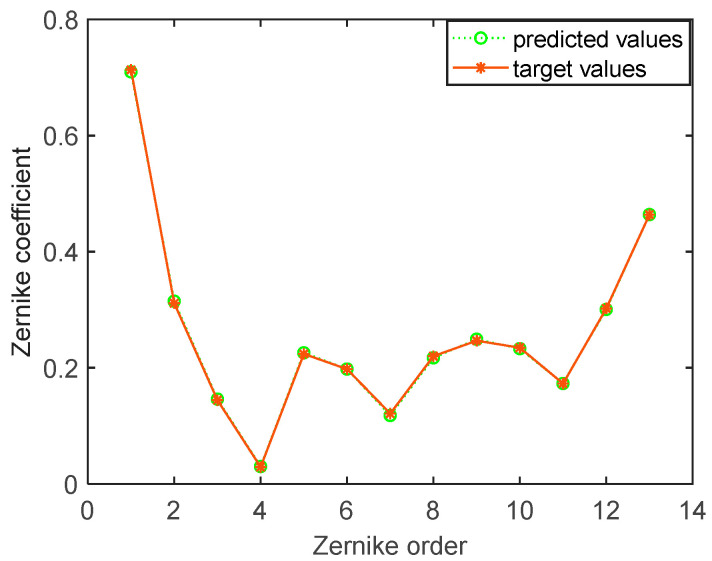
The predicted result of the ELM model.

Then we employ the trained ELM model to predict Zernike mode coefficients of the generated random wavefront, as shown in Figure 6. The condensing beam system of WFS is the Cassegrain structure, and the incident aperture is annular, as shown in Figure 6b,c. In the current sample, it can be obviously seen that WFS cannot fully detect the random wavefront aberration, and some sub-apertures have no light spot or weak intensity light spot. Figure 7 shows that the predicted result of the ELM model. When the intensity of the incident wavefront is non-uniform, the ELM model can predict the accurate Zernike mode coefficients. The MSE is 3.0718 × 10^−6^.

**Figure 8:**
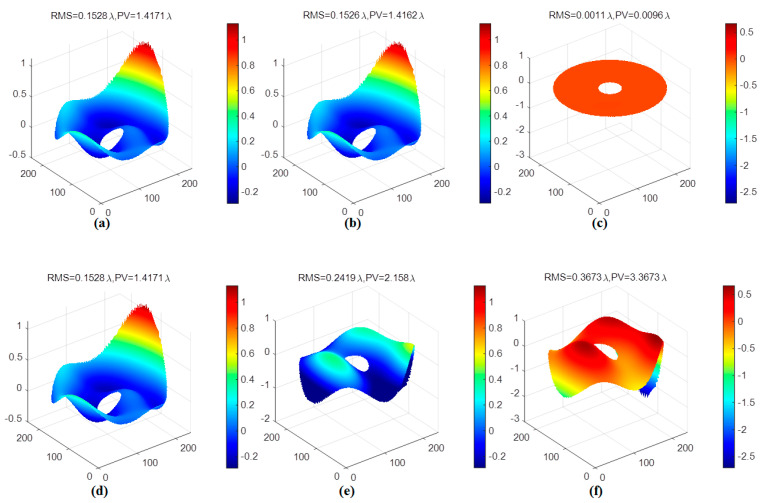
(**a**) The incident wavefront. (**b**) The restored wavefront by the ELM model. (**c**) The residual wavefront of the ELM model. (**d**) The incident wavefront. (**e**) The restored wavefront by the classical modal algorithm. (**f**) The residual wavefront of the classical modal algorithm.

**Figure 9:**
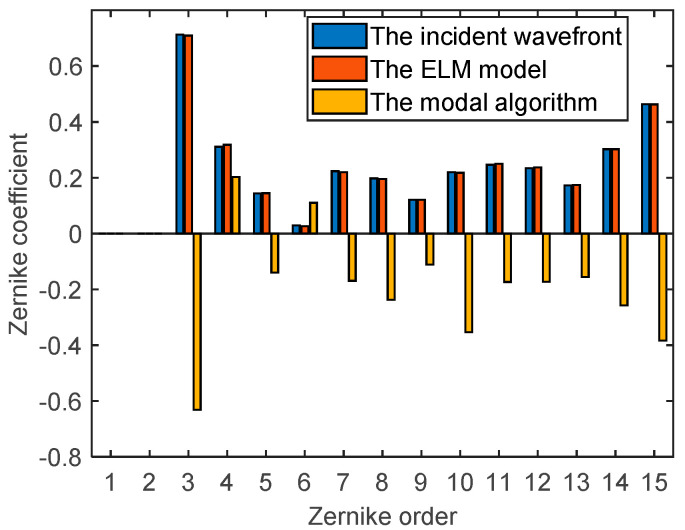
Wavefront restoration Zernike coefficients.

The generated random wavefront in Figure 6 is restored by Zernike modes coefficients which are obtained from the classical modal algorithm and the trained ELM model, respectively. The restored wavefront and residual wavefront are shown in Figure 8, respectively. According to Figure 8, the root mean square (RMS) and peak-valley value (PV) of the residual wavefront of the ELM model are 0.0011λ and 0.0096λ, respectively. The RMS and PV of the residual wavefront of the modal algorithm are 0.3673λ and 3.3673λ, respectively. Through the wavefront restoration comparison of the two methods, the ELM model has smaller RMS and PV of the residual wavefront and higher wavefront restoration accuracy. The first 15 order Zernike coefficients, respectively, predicted (excluding the first two order: Piston *a*_1_ and tilt *a*_2_) by the classical modal algorithm and the trained ELM model are shown in Figure 9. The prediction result of the proposed ELM model is significantly more accurate than the modal algorithm, according to Figure 9. There is a big discrepancy between Zernike coefficients obtained by the classical modal algorithm and the target values in Figure 9. The main reason is that the wavefront phase information detected by WFS is insufficient and inaccurate under the non-uniform intensity distribution.

**Figure 10:**
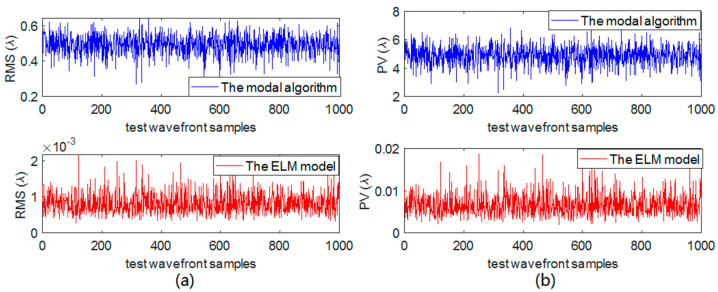
(**a**) The RMS value of the residual wavefront. (**b**) The PV value of the residual wavefront.

**Figure 11:**
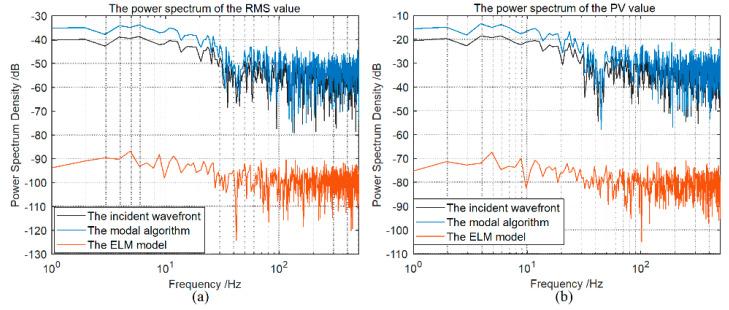
(**a**) The RMS power spectrum of the residual wavefront. (**b**) The PV power spectrum of the residual wavefront.

To further study the generalization of the ELM model, we use all the different light intensity distributions to expand the number of test samples to 1000 sets of wavefronts with the dynamic non-uniform intensity. The mean RMS and PV values of the incident wavefronts are 0.2831λ and 2.8501λ, respectively. The RMS and PV values of the residual wavefront of these two methods are shown in Figure 10. The mean RMS and PV values of the residual wavefront of the ELM model are 0.0082λ and 0.0066λ, respectively, while that of the modal algorithm are 0.4396λ and 4.8990λ, respectively. According to Figure 11, the RMS and PV power spectrum curve of the residual wavefront, based on the ELM model, is obviously smaller than the modal algorithm. Therefore, compared with the modal algorithm, the proposed ELM model can achieve much higher precision wavefront restoration under the dynamic non-uniform intensity distribution.

## 4. Conclusions

When the intensity of the laser presents dynamic non-uniform distribution, some sub-aperture light spots of WFS may have an irregular shape or weak intensity. The linear regression relationship between Zernike mode coefficients of the wavefront and the local slopes measured by WFS is no longer well satisfied. In this situation, it is difficult for the classical modal algorithm to restore accurate Zernike mode coefficients. The ELM model is proposed to establish the complex corresponding linear relationship between Zernike mode coefficients and the local slopes measured by WFS in the above cases. The simulation results prove that the proposed ELM model can restore the wavefront phase more accurately than the classical modal algorithm with fewer sub-apertures under the dynamic non-uniform intensity distribution. This improvement is beneficial to the applications where high precision wavefront restoration is necessary for the high energy and power laser beam purification system.

## Figures and Tables

**Table 1:** The key parameters of the simulations.

Parameters	Value
Wavelength	1064 nm
Numbers of micro-lens	10 × 10
Focal length of micro-lens	21.7 mm
Valid sub-aperture	76
Numbers of pixel in each sub-aperture	24 × 24 pixels
Pixel size	14 μm
Sampling frequency of WFS camera	1000 Hz

**Table 2:** The influence of different activation functions in the hidden layer on the predictive accuracy of the ELM model.

Activation Function	MSE
softplus	2.9489 × 10^−6^
Relu	2.6361 × 10^−5^
sig	7.5124 × 10^−5^
tanh	4.3081 × 10^−4^
sin	0.0024
hardlim	0.0015
RBF	0.0110
